# Role of erector spinae plane block in controlling functional abdominal pain: Case reports

**DOI:** 10.1097/MD.0000000000027335

**Published:** 2021-10-01

**Authors:** Kyudon Chung, Seung Tae Choi, Eun Hwa Jun, Se Gyu Choi, Eung Don Kim

**Affiliations:** a Department of Anesthesiology and Pain Medicine, Daejeon St. Mary's Hospital, College of Medicine, The Catholic University of Korea, Seoul, Korea, Jung-gu, Daejeon, Republic of Korea; b Department of Anesthesiology and Pain Medicine, St. Vincent's Hospital, College of Medicine, The Catholic University of Korea, Paldal-gu, Suwon-si, Gyeonggi-do, Republic of Korea.

**Keywords:** abdominal pain, erector spinae plane block, ultrasound

## Abstract

**Rationale:**

Functional abdominal pain is an intractable medical condition that often reduces quality of life. Celiac plexus block is a representative intervention for managing intractable abdominal pain. However, celiac plexus block can be technically difficult to perform and carries the risk of potential complications. During erector spinae plane block (ESPB), the injectate can enter the paravertebral space and reach the sympathetic chain. If local anesthetics spread to the sympathetic chain that supplies fibers to the splanchnic nerve, abdominal pain theoretically could be reduced.

**Patient concerns:**

Three patients suffered from abdominal pain of unknown cause, and no medical abnormalities were found in various examinations.

**Diagnosis:**

As a result of collaboration with related medical departments, the abdominal symptoms of the patients were suspected to be functional abdominal pain.

**Interventions:**

We successfully controlled symptoms by performing ESPB at the lower thoracic level in 3 patients with functional abdominal pain.

**Outcomes:**

After the procedure, the patients’ abdominal pain improved significantly over several months.

**Lessons:**

We suggest that lower thoracic ESPB could be an option for management of functional abdominal pain.

## 1 Introduction

Abdominal pain is one of the most commonly experienced symptoms reported throughout the human lifespan, accounting for approximately 5% of annual emergency department visits.^[1]^

This type of pain typically is organic in origin. However, other functional causes must be considered. Functional abdominal pain is difficult to diagnose and treat.^[2,3]^ Sympathetic blocks, such as celiac plexus blocks, can be applied to control chronic abdominal pain.^[4]^

Erector spinae plane block (ESPB) has been used in various pain conditions since its introduction in 2016.^[5]^ The analgesic effect of ESPB is believed to be achieved when local anesthetics (LA) enter the paravertebral space through the costotransverse foramen or intertransverse connective tissue complexes. Thoracic ESPB was effective for visceral pain as well as abdominal wall pain control following abdominal surgery.^[6]^ However, the use of ESPB for management of abdominal pain not related to surgery, such as functional abdominal pain, has yet to be reported.

In this article, we describe our clinical experiences using ESPB to control chronic functional abdominal pain in non-surgical conditions.

## 2 Case presentation

### 2.1 Method of intervention

For continuous ESPB, the patient was placed in a prone position, and a high-frequency linear probe (12–15 MHz) was placed longitudinally on the left transverse process (TP) at the T11 level.

**Figure 1:**
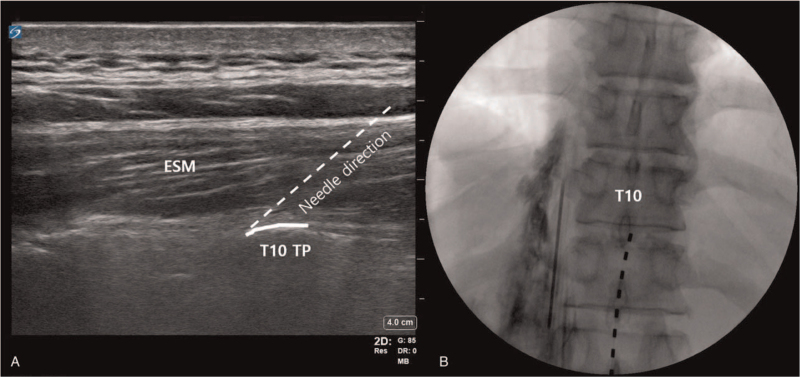
Continuous ESPB of patient 1. The dotted line indicates the needle direction, in a caudal to cephalad direction (A). Spreading between T9 and T12 levels was confirmed by injecting 2 mL of contrast medium (B). ESM = erector spinae muscle, ESPB = erector spinae plane block TP = transverse process.

After identifying the TP, an 18-gauge Tuohy needle was inserted to contact the TP using in-plane techniques. After bone contact, hydrodissection with 1 mL of saline was used to confirm the spread between the TP and the erector spinae muscle (Fig. 1A). A 20-gauge epidural catheter was inserted through the Tuohy needle, and the catheter tip was placed at the T10 level. Placement was confirmed by injecting 2 to 3 mL of contrast and verifying spread from the T9 to T12 level (Fig. 1B). Next, 10 mL of 0.5% lidocaine was injected through the catheter, and 0.187% ropivacaine was infused continuously at a rate of 2 mL per hour.

For single ESPB, a 22-gauge Tuohy needle was inserted using the same technique as performed in continuous ESPB. After bone contact with TP of the target level, hydrodissection with 1 mL of saline was used to confirm the spread between the TP and the erector spinae muscle. Then, 15 mL of 0.5% lidocaine was injected.

### 2.2 Patient 1

A 59-year-old male patient who suffered from neuropathic pain after a traffic accident 6 years before. The patient had been diagnosed with right lower extremity complex regional pain syndrome (CRPS) type 1 in accordance with the International Association for the Study of Pain diagnostic criteria and had been treated with various medications, including anticonvulsants, antidepressants, and opioids. He had previously undergone spinal cord stimulation (SCS) implantation 2 years previous.

One year ago, the patient began to complain of left-sided abdominal pain. There were no abnormal findings upon physical examination, and there were no signs of infection at the SCS battery implantation site. A thorough examination of the abdominal wall did not reveal any neuropathic features such as allodynia or hyperalgesia.

There were no gastrointestinal symptoms, such as diarrhea and constipation, and the patient underwent gastroscopy, colonoscopy, abdominal x-ray, and abdominal computerized tomography (CT), which were all normal. Finally, we received a reply from the department of gastroenterology that functional abdominal pain was suspected based on Rome Foundation Diagnostic Algorithms.^[2,3]^

An ESPB at the T10 level on the left side was applied to control the patient's unexplained functional abdominal pain. After a single ESPB, The intensity of abdominal pain of the patient decreased from the numeric rating scale (NRS) 8 to 3, and this effect lasted for about a day. Continuous ESPB with a catheter was chosen as a long-term method of pain control. The procedure was performed after receiving written informed consent from the patient, and written informed consent was obtained from the patient to publish the case study.

During catheterization, the patient reported approximately 80% improvement in abdominal pain. Catheterization was maintained for two weeks, and there were no side effects associated with the procedure. After catheter removal, improvement in abdominal pain was maintained for about 6 months.

### 2.3 Patient 2

A 31-year-old female patient on hemodialysis for diabetes mellitus (DM) and chronic renal failure was referred to a pain center from the department of gastroenterology for control of abdominal pain lasting for 2 years. She also exhibited no specific findings in any medical evaluation such as gastroscopy, colonoscopy, abdominal x-ray, and abdominal CT. The endocrinologist and nephrologist stated that her abdominal pain was not related to the underlying conditions.

The pattern of abdominal pain was deep in the epigastric region, and the patient complained that the pain intensity was higher than NRS of 9. No neuropathic features were observed in the abdominal wall. Based on the same diagnostic criteria as Patient 1, abdominal symptoms of the patient were also considered functional abdominal pain.

Since the glomerular filtration rate of the patient was reduced to 20.19 mL/min/1.73 m^2^, it was difficult to prescribe neuropathic medications such as gabapentioids. Neuroaxial blocks such as epidural block or paravertebral block were difficult due to general weakness of the patient. We decided to perform ESPB at the T7 level on both sides to control the patient's abdominal pain. Thirty minutes after the procedure, the patient's abdominal pain decreased to NRS 3. At the return visit two weeks later, she complained that the abdominal pain had increased to NRS 5. A second ESPB was conducted bilaterally at the T7 level using the same technique. After another 2 weeks, the procedure was repeated, and the patient's abdominal pain remained in the NRS 2-3 range.

### 2.4 Patient 3

A 34-year-old female patient with no specific medical history was referred to the pain center after going through the gastroenterology department for epigastric pain that started 4 months before.

The patient expressed the abdominal pain as a feeling of tearing in a deep area. There were no gastrointestinal symptoms other than abdominal pain. There were no specific findings in various medical examinations, and there was no response to medications such as proton pump inhibitors and prokinetic drugs. As a result, the gastroenterologist suggested that the patient's symptoms were functional abdominal pain based on same diagnostic criteria as Patient 1 and 2. The patient underwent a single ESPB at both T7 levels. Approximately 30 minutes after the procedure, the intensity of the patient's abdominal pain decreased from NRS 7 to 2. At a questionnaire 6 months after the procedure, the patient-reported resolution of abdominal pain.

In all 3 patients, any specific side effects related to the procedure were not observed.

## 3 Discussion

Functional abdominal pain is an intractable medical condition that often reduces quality of life.^[2,3]^ Celiac plexus block (CPB) or splanchnic nerve block is a representative intervention for managing intractable abdominal pain. The celiac plexus receives innervation from the greater, lesser, and least splanchnic nerves, which receive fibers from sympathetic chains located from the T5 to the T12 levels.^[4]^ The needle should penetrate deeply into the space around the vertebral body when performing splanchnic nerve block or CPB. This procedure can make the patient uncomfortable and carry risks, such as kidney injury. In addition, the target points of the needle tip in splanchnic nerve block or CPB are difficult to approach for catheterization for continuous LA infusion.

**Figure 2:**
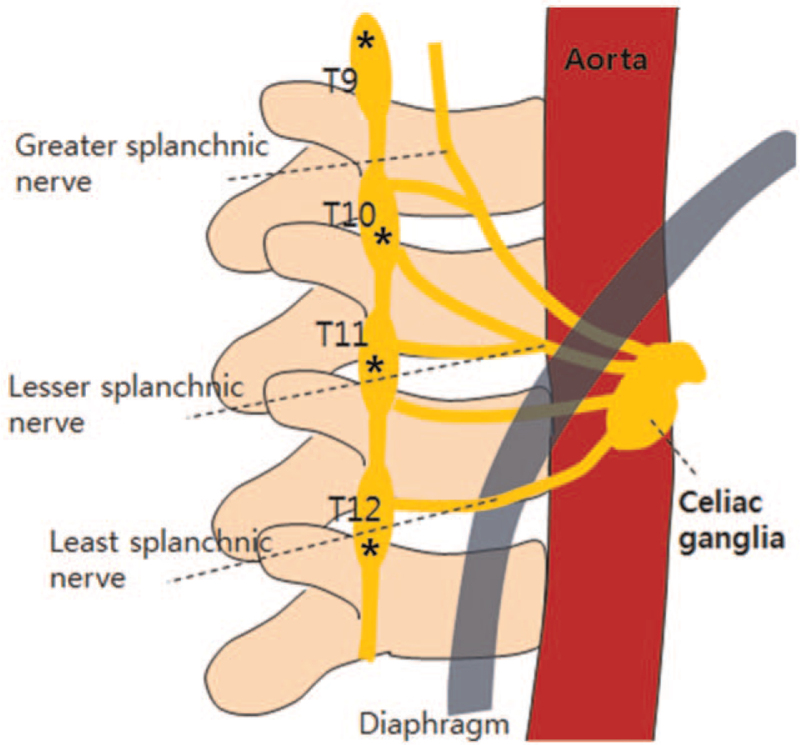
Schematic diagram of greater, lesser, and least splanchnic nerves from paravertebral sympathetic chains to the celiac plexus. During ESPB, if LA spread to the sympathetic chain (asterisk) that supplies fibers to the splanchnic nerve, these injectates theoretically reduce abdominal pain. ESPB = erector spinae plane block. LA = local anesthetics.

In contrast, ESPB is a very simple and relatively safe technique. The target interfascial plane in ESPB is relatively safer than a neuroaxial location, such as the epidural space, even for continuous LA infusion. During ESPB, the injectate can enter the paravertebral space and reach the sympathetic chain.^[5–7]^ Therefore, if LA spreads to the sympathetic chain that supplies fibers to the splanchnic nerve, it theoretically can reduce abdominal pain (Fig. 2). Many studies have reported the effectiveness of ESPB to control abdominal pain after abdominal surgery. Use of ESPB as a sympathetic block in upper-extremity CRPS has been previously reported.^[7]^

Functional abdominal pain can be caused by complex factors and has been reported to occur with increased pain sensitivity.^[8]^ CRPS is a situation where sensitivity to pain is increased and multiple medications are used in combination. Medical conditions such as DM and chronic renal failure might also involve increased pain sensitivity.

In particular, if there is general weakness in conditions accompanied by underlying diseases, it might be difficult to implement neuroaxial blocks such as an epidural block or even paravertebral block. If ESPB is conducted using LA of low concentration, the risk of cardiovascular complications could be reduced.

This case report is the first description of ESPB application for functional abdominal pain associated with non-surgical conditions. We suggest that lower thoracic ESPB could be an option for management of abdominal pain without a specific cause. Further research is needed to confirm our findings.

## Author contributions

**Conceptualization:** Kyudon Chung, Eung Don Kim.

**Data curation:** Kyudon Chung, Seung Tae Choi, Eun Hwa Jun, Se Gyu Choi, Eung Don Kim.

**Formal analysis:** Kyudon Chung, Seung Tae Choi, Eun Hwa Jun, Se Gyu Choi, Eung Don Kim.

**Investigation:** Seung Tae Choi, Eun Hwa Jun, Se Gyu Choi, Eung Don Kim.

**Methodology:** Eung Don Kim.

**Software:** Eung Don Kim.

**Supervision:** Eung Don Kim.

**Validation:** Seung Tae Choi, Eung Don Kim.

**Visualization:** Eung Don Kim.

**Writing – original draft:** Kyudon Chung, Eung Don Kim.

**Writing – review & editing:** Kyudon Chung, Eung Don Kim.
